# Evaluating the Accuracy and Repeatability of Mobile 3D Imaging Applications for Breast Phantom Reconstruction

**DOI:** 10.3390/s25154596

**Published:** 2025-07-24

**Authors:** Elena Botti, Bart Jansen, Felipe Ballen-Moreno, Ayush Kapila, Redona Brahimetaj

**Affiliations:** 1 Department of Electronics and Informatics (ETRO), Vrije Universiteit Brussel (VUB), Pleinlaan 2, B-1050 Brussels, Belgium; 2 IMEC, Kapeldreef 75, B-3001 Leuven, Belgium; 3 Robotics & Multibody Mechanics (R&MM) Research Group, Vrije Universiteit Brussel, B-1050 Brussels, Belgium; 4 Flanders Make, B-1050 Brussels, Belgium; 5 Department of Plastic, Reconstructive and Aesthetic Surgery, Brussels University Hospital (UZ Brussel), Vrije Universiteit Brussel (VUB), Laarbeeklaan 101, B-1090 Brussels, Belgium

**Keywords:** 3D mesh reconstruction, 3D imaging technologies, mobile 3D scanning, breast reconstructive surgery, plastic surgery

## Abstract

Three-dimensional imaging technologies are increasingly used in breast reconstructive and plastic surgery due to their potential for efficient and accurate preoperative assessment and planning. This study systematically evaluates the accuracy and consistency of six commercially available 3D scanning applications (apps)—Structure Sensor, 3D Scanner App, Heges, Polycam, SureScan, and Kiri—in reconstructing the female torso. To avoid variability introduced by human subjects, a silicone breast mannequin model was scanned, with fiducial markers placed at known anatomical landmarks. Manual distance measurements were obtained using calipers by two independent evaluators and compared to digital measurements extracted from 3D reconstructions in Blender software. Each scan was repeated six times per application to ensure reliability. SureScan demonstrated the lowest mean error (2.9 mm), followed by Structure Sensor (3.0 mm), Heges (3.6 mm), 3D Scanner App (4.4 mm), Kiri (5.0 mm), and Polycam (21.4 mm), which showed the highest error and variability. Even the app using an external depth sensor (Structure Sensor) showed no statistically significant accuracy advantage over those using only the iPad’s built-in camera (except for Polycam), underscoring that software is the primary driver of performance, not hardware (alone). This work provides practical insights for selecting mobile 3D scanning tools in clinical workflows and highlights key limitations, such as scaling errors and alignment artifacts. Future work should include patient-based validation and explore deep learning to enhance reconstruction quality. Ultimately, this study lays the foundation for more accessible and cost-effective 3D imaging in surgical practice, showing that smartphone-based tools can produce clinically useful scans.

## 1. Introduction

Three-dimensional (3D) surface imaging has become a valuable tool in plastic surgery, particularly in aesthetic procedures such as breast augmentation and facial contouring. It surpasses traditional two-dimensional (2D) photography by providing comprehensive spatial data. This capability significantly enhances preoperative analysis and surgical planning accuracy [1]. While its use in oncoplastic and reconstructive breast surgery—especially in cancer contexts—remains limited due to current constraints in accuracy and clinical validation, 3D imaging holds potential for future integration. In standard clinical practice, surgeons primarily rely on radiographic images such as mammograms/MRI scans for tumor localization and surgical planning [2]. However, these images are usually acquired with patients in standing (mammography) or prone (MRI) positions, which do not reflect the supine posture typically used during surgery. Additionally, breast compression during mammography, along with differences in patient positioning in both modalities, can complicate the accurate translation of imaging data into surgical scenarios. Also, these discrepancies can compromise tumor removal and negatively affect reconstructive outcomes, highlighting the need for improved (position-consistent) preoperative planning.

Various techniques such as structured-light scanners, laser scanners, and stereophotogrammetry have emerged as viable solutions for capturing the complex geometry of the breast. Structured-light and stereophotogrammetry systems have demonstrated sub-millimeter (mm) precision and reliability (with typical errors well under 1 mm in controlled settings) [3,4,5], but their clinical adoption remains limited. The trade-offs lie in cost, complexity, and capture speed/time. For instance, multi-camera photogrammetry enables rapid acquisitions but at high cost and complexity, whereas structured-light and laser scanners, though portable, require a steadier subject due to longer scan durations and have limitations in mesh quality. In response, recent developments in mobile scanning devices and smartphone-based applications offer a promising balance of accuracy, reproducibility, and accessibility [6].

One notable device is Structure Sensor, an infrared depth camera attachable to iPads. Oranges et al. [7] evaluated it on an iPad Pro by scanning a rigid female torso phantom alongside two commercial system (Canfield Vectra M5 and Artec Eva). Analysis of breast measurements (distances between anatomical landmarks and surface areas) showed no significant differences, indicating that low-cost mobile scanners can match the accuracy of high-end systems in controlled settings. This finding supports the conclusion that, for a controlled model, a mobile infrared scanner can be as reliable as industry-standard 3D imaging systems in capturing breast morphology. Koban et al. [8] compared an affordable handheld scanner (Intel RealSense) against a multi-camera photogrammetric system in 42 patients. Breast measurement showed good-to-excellent correlation between the tested devices, with surface deviations around 1.6–1.8 mm root-mean-square (RMS) error and a reproducibility of 0.64 mm RMS.

Beyond these, modern smartphones further extend mobile scanning capabilities through two main approaches: photogrammetry apps and LiDAR-based scanning. Photogrammetry apps reconstruct 3D meshes by stitching together multiple images, while newer devices equipped with LiDAR sensors allow direct depth capture. One recent study [9] compared smartphone-derived 3D breast volumes (3D Scanner App was used) with MRI-based volumes (MRI being a gold standard for volume measurement) in 22 women. While small systematic offsets were observed, the results demonstrated that smartphone scanning can yield reasonably accurate volume estimates at low cost. Han et al. [10] evaluated a smartphone LiDAR workflow in 25 patients, focusing on clinically relevant (linear) breast measurements. Relative errors were low (approximately 1.4% for notch-to-nipple and 3.5% for nipple-to-midline distances) with excellent inter-rater reliability (ICC—0.92). Notably, the learning curve was short, with clinicians achieving proficiency after only a few scans. All these findings suggest that portable, affordable solutions could achieve clinically acceptable accuracy in breast imaging, provided their limitations are well understood and characterized.

While smartphone-based 3D scanning apps provide convenient solutions, many use proprietary reconstruction algorithms that often rely on deep learning (DL) methods, the specifics of which remain undisclosed. In contrast, recent academic research has developed open-source DL frameworks capable of reconstructing detailed meshes from sparse inputs. For example, Occupancy Networks [11] accurately learn continuous shape representations from single images or sparse point clouds and they have been validated on large synthetic datasets, such as ShapeNet (over 50,000 synthetic 3D models) [12]. Pixel-Aligned Implicit Functions (PIFu) [13] reconstruct detailed clothed human meshes from one or more RGB images, showing robustness to occlusions by aligning pixel-level features with learned shape priors. Alternative approaches such as convolutional mesh regression [14] and graph-based approaches like Pixel2Mesh [15] predict mesh vertex positions or deform template meshes from single images, achieving high accuracy on real-world human datasets. Specifically in breast reconstruction, recent open-source DL developments remain largely experimental or in early clinical stages. For instance, Weiherer et al. [16] introduced an implicit neural breast shape model (iRBSM), trained on 168 photogrammetry-based 3D scans, which reconstructs detailed breast meshes from single 2D photos. Their workflow uses a state-of-the-art monocular depth estimator (Depth Anything V2) to generate depth maps, reprojected into 3D using known camera intrinsics, followed by model fitting to the resulting point cloud. Duarte et al. [17] proposed a CNN-based system for real-time reconstruction, validated on synthetic data with a mean surface error of approximately 3.9 mm. Commercial tools like Crisalix also use DL algorithms (despite proprietary) to generate 3D breast models from photographs for preoperative planning. However, all these methods face key limitations, including limited datasets, difficulty achieving clinical-grade accuracy, and the need for real-world validation.

Given this context, the clinical utility and practical capabilities of currently available commercial 3D scanning applications still require rigorous evaluation. The primary objective of our study was to evaluate and quantify the performance of a selected set of current applications—3D Scanner App, Heges, Polycam, SureScan, Kiri and Structure Sensor—in reconstructing 3D breast meshes, using manual measurements as the reference for validation. A mannequin phantom fitted with silicone breasts was employed to maintain controlled conditions. Specifically, we focused on the following:

1. **Overall reconstruction accuracy across applications:** Assessed by comparing manually measured anatomical distances on the phantom with corresponding digital measurements extracted from 3D meshes, aggregated across all scans.
2. **Intra-trial and operator-related variability:** Evaluated by analyzing repeated scans per app and comparing error consistency across trials and between two independent operators.
3. **Anatomical region-specific error patterns:** Analyzed by examining reconstruction accuracy across different anatomical distances to identify which regions are more prone to distortion or variability.
4. **Visual and practical challenges:** Documented through qualitative inspection of reconstructed meshes.

By systematically addressing these objectives, this study provides insights into the reliability, accuracy and practical limitations of current 3D reconstruction applications for breast mesh generation using a phantom model.

## 2. Materials and Methods

### 2.1. Phantom Setup

**Figure 1:**
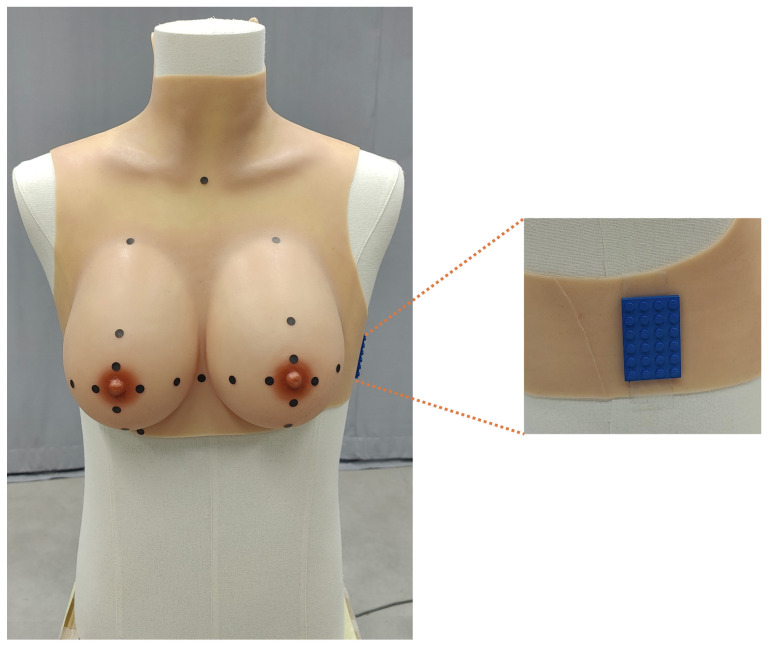
Illustration of the breast phantom with markers and the Lego brick used for calibration.

The dataset used in this study was generated using a silicone-based female torso phantom, placed on a mannequin to ensure realistic positioning during the scanning process. To address potential scaling inaccuracies encountered with some applications, a Lego block with uniform dimensions of 3.2 cm × 4.8 cm and 90° angles was placed on the left side of the phantom. The object served as a reference for scale calibration [18]. All scans were performed under controlled lighting and stable environmental conditions to minimize variability (Figure 1).

### 2.2. Manual Anthropometric Measurements

**Figure 2:**
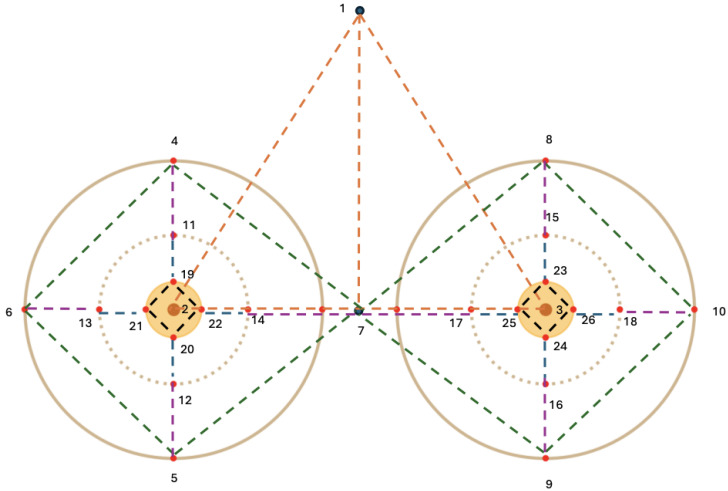
Distances measured on the breast phantom. Dotted lines represent measured distances, while points represent fiducial marker locations.

Physical markers (adhesive black stickers) were strategically placed on key landmarks including the nipples, sternum, breast corners, nipple–areola boundaries, and peripheral nipple areas (to enable precise small-distance measurements). Some of these landmarks (i.e., nipples, sternum, breast corners) were selected based on their common use as reference points in aesthetic and reconstructive breast surgery to assess breast symmetry, contour, and anatomical positioning [19]. In particular, the nipples and sternum serve as primary orientation points in surgical planning, while nipple–areola boundaries and breast corners support detailed evaluation of local distortions. To enhance spatial resolution in critical regions, our acquisition setup included additional peripheral nipple(–areola) markers, specifically aimed at capturing short-range deformations around high-curvature areas and enabling precise measurement of localized distortions. Linear distances between selected markers were manually measured using a digital caliper (precision: 0.1 mm) by two independent operators. The caliper was positioned carefully between the centers of marker pairs to reflect accurate linear distances. To mitigate measurement bias, the mean value of the manual measurements (between the two operators) was defined as the ground truth. The inter-operator absolute difference across all measurements was consistently low, with a mean error of 0.88 mm across all distances. Figure 2 provides an overview of all distances measured on the phantom.

### 2.3. Selection and Overview of 3D Scanning Applications

The 3D scanning apps used in this study were selected through a two-step process. First, potential apps were identified via App Store/Play Store searches, beginning with well-established scanning apps (such as the Scanner app developed for the Structure Sensor) and expanding to additional apps suggested by the app stores. Their suitability for medical and anthropometric use was further evaluated via academic searches on Google Scholar (search query: ‘[App name] + breast reconstruction’), specifically targeting their relevance in breast reconstruction. The shortlisted apps we used were Heges, Polycam, Kiri Engine, 3D Scanner App and SureScan. In addition to these mobile apps, we also assessed the Structure Sensor—a widely used external device designed to capture 3D data. It acquires depth information via an infrared sensor while relying on the iPhone’s built-in RGB camera for color capture. The use of the Structure Sensor requires calibration, which was carefully performed following the step-by-step instructions provided during the calibration process. A detailed summary of the scanning configurations and export settings that we used for each application is provided in Appendix A.

### 2.4. Digital Measurements Using Blender

Digital measurements were performed using Blender, an open-source 3D modeling software. Blender’s built-in measurement tools enabled precise and reproducible assessment of linear distances between annotated markers on the reconstructed digital breast models. Specifically, measurements were taken between the automatically computed centers of the black markers, analogous to the manual measurements. These digital measurements were directly compared against the established manual (caliper) measurements, thereby providing quantitative evaluation of the reconstruction accuracy of each scanning application.

### 2.5. Methods

To validate the performance of each scanning app, we conducted software-based measurements between anatomical landmarks on the reconstructed meshes. Digital measurements were performed by a single operator.

Scans were acquired by two operators, each performing three independent scans per application, resulting in six trials per app. All scans were performed under same controlled conditions to minimize environmental variability and ensure reproducibility. Prior to selecting the final set of applications and parameter configurations used in this study, we conducted preliminary tests across a broader range of applications and parameter setups. This initial exploration allowed us to identify the optimal parameter configurations for each application, balancing reconstruction accuracy, marker visibility, and visual quality. Applications that consistently failed to reconstruct markers or produced poor-quality meshes were excluded from further analysis. This iterative process refined our experimental setup to include only the most promising tools for systematic evaluation. All scans were performed using an iPad Pro (3rd generation) running iPadOS 18.3 (22D63).

## 3. Results

To address our study objectives, the results are structured into four subsections as outlined below:

### 3.1. Overall Reconstruction Accuracy Across Applications

**Figure 3:**
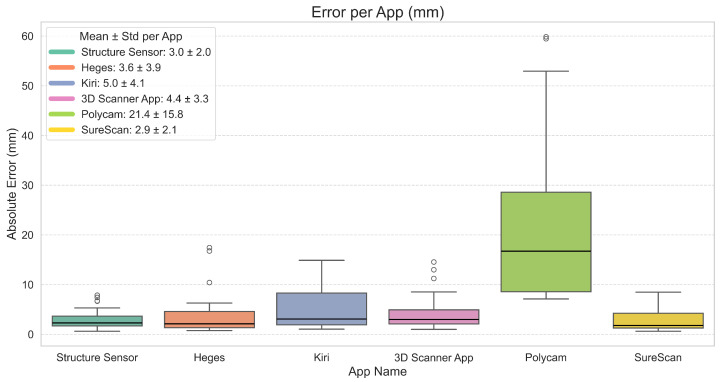
Distribution of absolute errors across all trials and landmark distances per app.

For each of the six trials per app, we computed the absolute error between each reconstructed and true landmark distance. Rather than averaging errors within each trial, we aggregated all individual measurement errors across all six trials (216 measurements per app) and visualized the resulting distributions using boxplots (Figure 3). This approach provides a granular view of both central tendency and variability. Averaging per trial would obscure within-scan variability and potentially mask outlier behavior. Aggregating all individual distance errors across all landmarks and repeated scans (the way we analyze it now), the boxplots capture the full range of performance, offering a clearer view of each app’s consistency and robustness. Low variability and few outliers in this context indicate good average performance and stability. The mean absolute error and standard deviation (std) were as follows: SureScan (2.9 ± 2.1 mm), Structure Sensor (3.0 ± 2.0 mm), Heges (3.6 ± 3.9 mm), 3D Scanner App (4.4 ± 3.3 mm), Kiri (5.0 ± 4.1 mm), and Polycam (21.4 ± 15.8 mm). For Heges, some distances consistently showed large errors, while most remained low. When all distances are combined, this large difference between errors causes the std to exceed the mean.

Using these error distributions, we first applied a Kruskal–Wallis H-test (chosen due to non-normality and unequal variances between apps) to assess whether reconstruction errors differed significantly between applications. The test revealed a strong effect of the application on error distribution (H = 84.35, $p<1.03\times 10^{-16}$), indicating that at least one app performed differently from the others. We further performed pairwise comparisons using Dunn’s post hoc test with Holm correction. Results showed that Polycam differed significantly from all other applications (*p* < 0.001), while no significant differences were observed among the remaining apps (all *p* > 0.1; see Appendix B).

### 3.2. Intra-Trial and Operator-Related Variability

**Figure 4:**
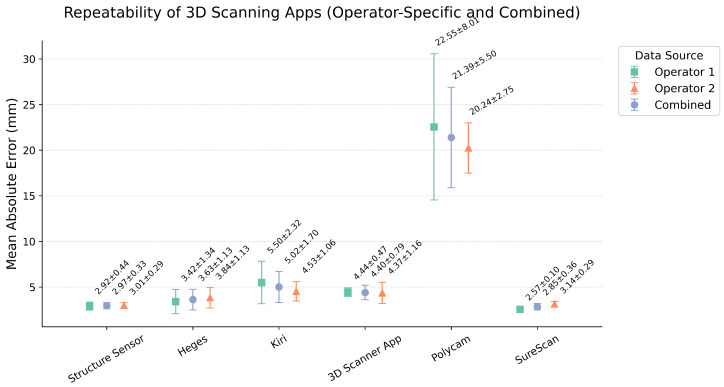
Repeatability of 3D scanning apps across multiple trials shown separately by operator and in combination. Results are shown separately for Operator 1, Operator 2, and combined. Mean differences between repeated trials are shown for each application, with error bars indicating the std across trials. Lower values reflect higher repeatability. SureScan and Structure Sensor demonstrated the most consistent performance, whereas Polycam exhibited markedly greater variability.

To assess the repeatability of each 3D scanning application, we summarized the previously computed landmark-level absolute errors by calculating the average error across all landmarks within each trial. This yielded a single performance value per trial, which was then averaged across trials to obtain the overall mean and std (both in mm) for each app (Figure 4). While this approach summarizes performance using average errors per trials, it does not account for the std of individual errors within each trial. Nonetheless, it provides a straightforward overview of each app’s repeatability across trials. Among all apps evaluated, Structure Sensor showed the highest repeatability with a mean error of 2.97 ± 0.33 mm, followed by SureScan (2.85 ± 0.36 mm), Heges (3.63 ± 1.13 mm), and 3D Scanner App (4.40 ± 0.79 mm). Kiri demonstrated moderate variability (5.02 ± 1.70 mm), while Polycam exhibited the largest inconsistency, with a mean intra-trial difference of 21.39 ± 5.50 mm. To assess whether operator variability influenced reconstruction accuracy, we conducted again the Kruskal–Wallis H-test using the full distribution of landmark-level error values grouped by operator. The analysis revealed no significant effect (all *p* > 0.15) of operator on the error distribution (H = 0.01, *p* = 0.93).

### 3.3. Anatomical Region-Specific Error Analysis

**Figure 5:**
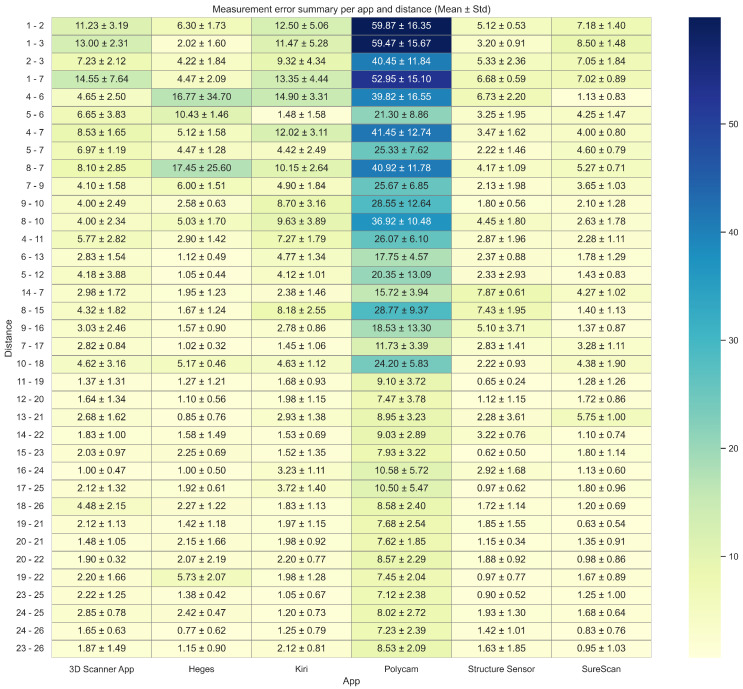
Heatmap of mean absolute measurement errors (± std) in mm across anatomical distances for each scanning application. Errors are computed from six independent trials per app.

To evaluate how reconstruction performance varies across different anatomical regions within each 3D scanning application, we analyzed landmark-to-landmark distance errors across repeated trials. An annotated heatmap (Figure 5) summarizes the mean ± std of reconstruction errors for each distance and application, aggregated over six trials. This visualization facilitates the identification of anatomical distances that are more error-prone within each application and highlights where measurement inconsistencies are most likely to occur.

When interpreted alongside the anatomical landmark schema (Figure 2), several patterns emerge that reflect the spatial complexity of the reconstruction task. A subset of distances exhibited consistently higher error magnitudes across multiple applications, particularly for Polycam. The most problematic were long-range or cross-quadrant measurements, which span medial-to-lateral or inferior-to-superior regions of the breast. These distances frequently exceeded 40–60 mm of error for Polycam, while other applications typically remained below 10 mm for the same landmarks. In contrast, shorter intra-quadrant distances—especially those surrounding the periareolar region (e.g., 22–23, 25–26, and 20–21)—showed low reconstruction error and minimal variability across trials for all platforms.

The std values shown in Figure 5 reflect trial-to-trial variability in reconstruction error for each distance and each app. These values offer insight into region-specific reconstruction consistency within each application. Kiri and 3D Scanner show higher variability compared to SureScan, Structure Sensor, and Heges. For most distances, SureScan, Structure Sensor, and Heges demonstrated low variability (typically < 2 mm). In contrast, Polycam not only yielded the highest mean errors but also showed elevated std for many distances.

A few distances, including 2–3 (nipple-to-nipple), 1–7, 1–2 and 1–3, exhibited relatively high variability across all applications. Conversely, periareolar distances (e.g., 19–22, 20–21, 20–22, 19–22) demonstrated both low error and low variability, reinforcing their reliability in scan-based measurements across platforms.

### 3.4. Visual Reconstruction (Practical Challenges)

**Figure 6:**
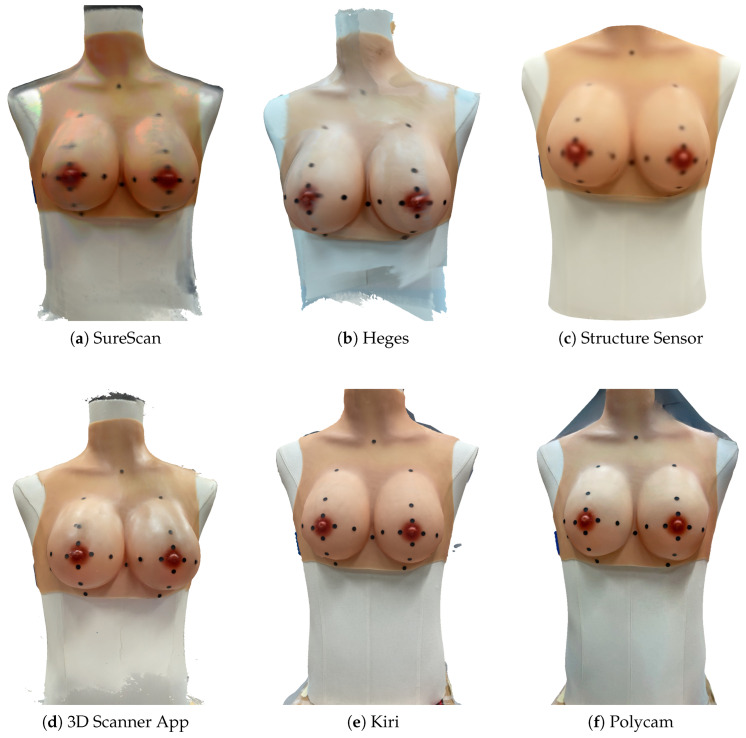
Most accurate mesh reconstructions from each app (lowest landmark error trial).

For each app, the trial with the lowest mean landmark error was selected to represent its most accurate output. These meshes are shown for illustrative purposes (Figure 6) to provide visual context regarding surface appearance and overall reconstruction quality per app. The examples also highlight differences in surface quality and anatomical detail: unclear boundaries of the markers (Figure 6a); lighting variations and unclear boundaries of the markers (Figure 6b); blurred reconstruction, with squared or blurred nipple–areola region (Figure 6c); marker duplication (Figure 6d); deformation of the breast volume (Figure 6e); deformation of the breast volume and inconsistent physical scale (Figure 6f). Despite these being the best scans for each app, (slight) visual and anatomical inaccuracies remain.

**Figure 7:**
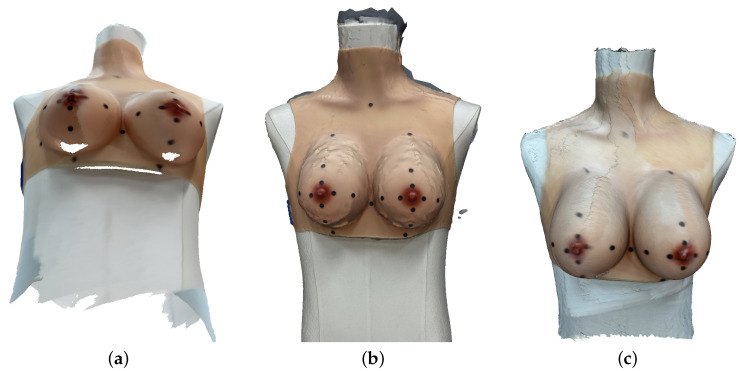
Examples of visual reconstruction issues across applications: (**a**) incomplete breast bottom surfaces observed with Heges; (**b**) surface irregularities in Kiri scans, despite low mean errors; (**c**) lighting and marker visibility issues, also shown in Heges.

Despite generally satisfactory reconstruction results, some practical challenges were encountered during the scanning process. Mesh quality was often influenced by the side from which the scan was initiated, with the first scanned side typically appearing less distorted. Incomplete geometry was frequently observed in the inferior region of the breasts, likely due to complex curvature and limited visibility (Figure 7a). Full 360° scans occasionally introduced alignment artifacts, likely resulting from errors in merging the front and back surfaces. Additionally, extended scanning sessions sometimes led to software instability or crashes. In some cases, despite low mean error values, the reconstructed meshes exhibited surface irregularities or unrealistic deformations in localized areas, emphasizing the importance of visual inspection and surface-based evaluation (Figure 7b). Reconstruction quality was also affected by ambient lighting conditions and marker visibility, which influenced both surface accuracy and marker tracking (Figure 7c). Although these represent the most accurate scans from each app, visual and anatomical inaccuracies remain and are illustrated in Figure 7. For some applications, colors were (slightly) inconsistent: the colors visible in the default app interface did not always match the appearance of the exported raw mesh if visualized in another software, suggesting that some degree of in-app color processing/enhancement may be applied during rendering. Moreover, even when using the same exported mesh, differences in color rendering and lighting between third-party software (e.g., Blender vs. MeshLab vs. Xcode) resulted in different visual impressions of the same model.

## 4. Discussion

The primary aim of this study was to systematically assess the accuracy and consistency of various commercially available mobile 3D scanning apps in generating anatomically accurate meshes of a female torso phantom with silicone breasts. While previous work has benchmarked individual apps or compared mobile scanners with high-end systems, a comprehensive evaluation of accuracy, repeatability and region-specific performance across multiple consumer solutions has so far been lacking. This paper addresses that gap by providing the first systematic assessment of six available mobile 3D scanning applications—SureScan, Heges, 3D Scanner App, Polycam, Kiri Engine, and Structure Sensor—using a controlled phantom model. Our findings provide a practical, evidence-based resource to help researchers and clinicians make informed decisions when selecting 3D scanning workflows that balance accuracy, cost, and real-world usability.

Among the tested applications, SureScan emerged as the best performer in terms of mean reconstruction accuracy, achieving a mean absolute error of approximately 2.9 mm. This is comparable to previously reported accuracies for high-end structured-light and stereophotogrammetry systems [3,4,7]. Structure Sensor and Heges also showed similar performance, with mean errors close to SureScan, supporting prior observations that LiDAR and infrared-based handheld scanners can be accurate [1]. However, the Kruskal–Wallis test and follow-up analyses revealed no statistically significant differences in accuracy between SureScan, Heges, Structure Sensor, Kiri, and 3D Scanner App. From a clinical perspective, this lack of significant difference may be seen as a strength: it suggests that several mobile apps can deliver comparable reconstruction accuracy, allowing users to choose based on workflow needs, cost, and device compatibility. Still, statistical similarity does not imply similar performance in practice. Polycam stood out with a much higher mean error of around 21.4 mm and large variability across scans. Interestingly, its reconstructions often appeared visually acceptable. The issue likely lies in its reconstruction method, which produces reasonable shapes but struggles with scaling. Without consistent scaling, measurements become unreliable. This entire limitation highlights an important point: visual quality alone does not guarantee quantitative accuracy.

Although this study did not include direct comparisons with clinical-grade systems like Canfield Vectra M5 and/or Artec Eva, the observed mean errors (e.g., 2.9 mm for SureScan) fall within clinically acceptable thresholds for surgical planning and breast symmetry assessment tasks (typically 3–5 mm) [3,7]. This suggests that mobile, camera-based tools may already offer sufficient accuracy for selected clinical applications, particularly where usability, cost, and repeatability are key considerations. This raises a broader question for the field: should future innovation focus more on hardware, on reconstruction methods, or ideally on both? Five of the six evaluated applications rely entirely on the iPad’s built-in RGB camera and depth estimation, with only Structure Sensor using an external depth sensor. This common imaging source enables a clearer comparison of reconstruction performance. Despite using similar input, the apps produced a wide range of mean errors (from 2.9 to 21 mm), indicating that differences in performance are largely driven by the reconstruction algorithms and post-processing techniques. Visual assessments reinforce this finding. As shown in Figure 6, all apps generated plausible-looking reconstructions, but each exhibited specific limitations. Structure Sensor produced smooth and realistic meshes with low error but tended to over-smooth surfaces, potentially hiding detail. However, when combining accuracy, consistency, and visual inspection, Structure Sensor appears to offer a high reliable and well-rounded performance. SureScan showed the highest accuracy but often lacked sharp visibility of fiducial markers and surfaces. Heges appeared sensitive to lighting, leading to surface noise in some scans. Both SureScan and 3D Scanner App occasionally duplicated markers. Kiri and Polycam showed volume distortions, with Polycam particularly affected by scaling issues despite coherent overall shapes. These findings suggest that performance depends not only on sensor quality or visual appeal but heavily on the reconstruction pipeline. Given that several apps using the same hardware delivered different (quantitative and qualitative) results, future improvements may be more effectively achieved through algorithmic enhancements (such as DL-based surface completion, mesh denoising, or improved marker detection) than through hardware upgrades alone. That said, a combination of software and hardware advancements may be necessary to fully overcome current limitations. If the strengths of individual approaches were integrated (e.g., Structure Sensor’s surface realism, SureScan’s accuracy, and more reliable marker handling), significantly improved results could be achieved at low costs.

Other analyses revealed specific anatomical patterns influencing reconstruction accuracy. Short-range distances, particularly those concentrated around periareolar regions, consistently exhibited high accuracy and low variability across most applications. This pattern is likely attributable to the relatively simpler surface geometry and the localized area of reconstruction, aligning with previous studies suggesting higher reliability of local morphological assessments using mobile scanning technologies [9,10]. In contrast, longer cross-quadrant measurements, encompassing medial–lateral and superior–inferior transitions, presented significantly greater reconstruction challenges across all platforms, notably in apps with lower algorithmic robustness, such as Polycam. This underscores the critical need for enhanced reconstruction algorithms capable of accurately capturing complex geometries/morphologies.

We also looked at how consistent each app was across repeated trials. SureScan and Structure Sensor again performed best, showing low variability between scans. Polycam, in contrast, was much less consistent, with high differences between repeated measurements. These results suggest that although some scanning apps deliver consistent results, others show some variability across repeated measurements, which may impact their reliability in clinical applications.

Operator-related variability was minimal. The Kruskal–Wallis test showed no significant differences between operators, suggesting that inter-operator variability had minimal impact on measurement performance across trials and applications. This is encouraging from a clinical perspective, as it means scanning can be performed reliably by different users, if a standard protocol is followed.

The heatmap in Figure 5 gives a detailed breakdown of errors by anatomical distances. While it may seem complex/dense at first, it allows users to identify which application yields more reliable distance measurements for specific clinical requirements. Periareolar distances were among the most accurate and consistent across all apps, likely because they involve small, local areas with relatively simple surface geometry. Cross-quadrant distances, especially those covering medial–lateral or superior–inferior directions, were more error-prone. These results indicate that while some applications maintain consistently low variability across repeated trials, others are more affected by spatial error fluctuations depending on scan region and landmarks. While we report absolute errors directly, future work could explore fuzzy logic-based uncertainty visualization techniques to help communicate confidence levels in distance estimations, particularly in regions prone to spatial error fluctuations [20].

We also observed several practical challenges during scanning. Mesh quality often depended on where the scan was started, with the first scanned side appearing less distorted. Some scans showed incomplete geometry at the bottom of the breasts, likely due to difficult viewing angles and complex curvature. Full 360° scans occasionally led to alignment issues, possibly caused by cumulative drift or loop closure errors. Even when overall errors were low, we sometimes found local surface deformations or irregularities. Lighting conditions and marker visibility also influenced surface accuracy. Moreover, the colors shown in-app did not always match the exported mesh when viewed in other software, likely due to in-app color processing. This introduces a source of variability in visual evaluation, emphasizing the need to standardize the rendering environment or interpret visual assessments with caution.

Our results suggest that mobile 3D scanning can already achieve clinically acceptable accuracy. Notably, a dedicated depth sensor such as the Structure Sensor is not strictly necessary to obtain high accuracy—apps like SureScan and Heges performed comparably well using only the iPad’s built-in sensors. However, the choice of application plays a critical role, as not all apps employ equally robust (qualitative) reconstruction algorithms. Even among apps with statistically similar performance, qualitative differences in repeatability and visual quality still matter.

The overall similarity in reconstruction accuracy (despite differences in app design and unknown internal implementations) prompted us to investigate whether these apps might rely on a shared underlying framework, specifically Apple’s ARKit. Since none of the applications publicly disclose their technical architecture, we analyzed their *.ipa* files (Apple’s binary package format for iOS apps) to identify potential API dependencies. These findings should be interpreted with caution, as they were not confirmed by the developers and the binaries were stripped of symbolic information, meaning internal method names and class structures were obfuscated. Nevertheless, they provided insights into potential dependencies. We found that Heges, Polycam, KIRI Engine, and 3D Scanner App all link to ARKit, while SureScan does not. For Heges, the internal logging evidence strongly suggests use of ARKit’s LiDAR features such as ARSession, ARPointCloud, and ARMeshAnchor for real-time reconstruction. For Polycam and KIRI, internal strings in their logs like ’sceneImage’ and ’meshAnchors’ suggest similar/possible conditional or runtime ARKit usage. The Structure Sensor app primarily relies on its proprietary depth hardware, with ARKit listed as an optional dependency not evidently used in our recordings.

Notably, SureScan (despite not using ARKit) achieved the lowest mean error, followed closely by ARKit-enabled apps like Heges, and 3D Scanner App. KIRI performed slightly worse, and Polycam, though also linked to ARKit, showed the highest error and variability. These findings suggest that while ARKit may provide a shared baseline for motion tracking and depth acquisition, its mere inclusion does not guarantee high quantitative and/or qualitative performance. Instead, performance appears to depend critically on how these tools are implemented and integrated into each app’s broader reconstruction pipeline. This reinforces the central role of software strategy and algorithmic design—beyond hardware and API choice—in determining overall reconstruction quality.

While our study focuses on static surface mesh reconstruction using phantom models, future clinical applications will require handling patient-specific challenges, such as soft tissue deformation, anatomical variability, and motion during scanning. DL-based reconstruction frameworks offer promising solutions, as they can learn statistical shape priors from large datasets and adapt to local anatomical variability, enabling more complete and anatomically coherent reconstructions even with missing or noisy input data [21,22]. In particular, combining spatial and temporal learning (which process both geometric structure and temporal evolution) has demonstrated strong performance in reconstructing dynamic anatomical textures. For example, STINR-MR [23] models 3D motion in cine-MRI, combining spatial and temporal implicit neural representations, while TransforMesh [24] uses mesh transformers to analyze long-term anatomical changes. Additionally, MedNeRF [25], a medically adapted Neural Radiance Field, enhances soft tissue reconstruction from limited/imperfect visual data.

Beyond improving reconstruction itself, biomechanical simulation methods such as finite element modeling (FEM) could serve as a complementary tool to validate reconstructed meshes. Multiscale FEM has already been used to simulate breast tissue deformation during surgery and wound healing, demonstrating realistic behavior when subjected to physiological loads [26]. Incorporating FEM-based validation in the pipeline would provide an additional layer of structural credibility—especially important for clinical applications like surgical planning, where mesh behavior under load is as critical as its shape.

Additionally, AI-based closed-loop scanning guidance systems could help reduce operator dependency by providing real-time feedback during acquisition (highlighting incomplete regions, poor surface coverage, or suboptimal scan angles). Similar AI techniques have already shown benefits in clinical imaging: for instance, AI-assisted ultrasound systems guide operators to capture optimal views and reduce variability between users [27]. Finally, fuzzy logic systems could potentially support uncertainty visualization by providing interpretable estimations of local reconstruction confidence, though their application to mesh error quantification remains exploratory. Although our pipeline focuses on surface mesh reconstruction, future multimodal frameworks could integrate non-optical sensing techniques such as impedance tomography or electromagnetic imaging, which have demonstrated promise in detecting internal breast tissue variations and deeper structural discontinuities [28,29].

Moreover, the field would benefit from open benchmarking initiatives that include shared datasets and standardized reporting of error distributions to enhance reproducibility and transparent comparison. Importantly, the ability of simple, smartphone-based tools to produce accurate 3D scans opens the door to more accessible and affordable workflows, especially in settings without access to high-end commercial systems. This also creates (new) opportunities for building datasets to train DL models for applications such as simulating postoperative breast morphology after lumpectomy or mastectomy. While these applications remain exploratory, they highlight the need for ethically sourced, anatomically diverse 3D data and robust validation before clinical use. Mobile scanning could eventually support not just documentation, but also personalized surgical planning and predictive modeling.

## 5. Study Limitations and Future Work

Our study has several limitations. First, the validation was performed using a single silicone breast phantom. While this setup ensured consistency and control, it does not reflect the full range of anatomical diversity encountered in clinical practice. Future evaluations should include phantoms with varying breast sizes, shapes, and skin tones to better generalize findings. Future work should also include scans of real patients, particularly individuals seeking aesthetic breast surgery (to assess performance under realistic clinical conditions). Second, the set of applications was not derived from a systematic or exhaustive review. Expanding the range of tested applications and applying more structured selection criteria would strengthen future analyses. Third, the study focused exclusively on phantom data. While this was necessary to eliminate variability from motion and soft tissue deformation, it limits the clinical applicability of the findings. Additionally, although this study thoroughly assessed geometric accuracy using landmark distances, we did not perform volumetric analysis of the reconstructed meshes. Such analysis would provide a valuable quantitative assessment of overall surface fidelity and will be a focus of future research. Lastly, our app evaluation did not include certain clinical platforms commonly used in aesthetic surgery, such as Vectra, Crisalix, Arbrealabs, and the Natrelle 3D Visualizer. Vectra was excluded due to its high cost, proprietary hardware, and limited accessibility. While Crisalix was initially considered, its AI-based reconstruction, optimized for real anatomy, produced unrealistic outputs on the phantom model, and hence, we excluded it from the list. Other apps like Arbrealabs and Mentor’s Breast Implant Simulator were not evaluated. In addition, these platforms typically do not provide access to raw mesh files, limiting our ability to perform detailed geometric comparisons. Nonetheless, given their clinical relevance, future studies in real subjects should consider including these systems.

## 6. Conclusions

This study provides the first systematic evaluation of six commercially available mobile 3D scanning applications (SureScan, Heges, 3D Scanner App, Polycam, Kiri Engine, and Structure Sensor) using a controlled silicone phantom model to assess their accuracy, repeatability, and practical limitations for breast reconstruction.

Key findings: (a) SureScan demonstrated the lowest mean absolute error (2.9 mm), followed by Structure Sensor (3.0 mm), Heges (3.6 mm), 3D Scanner App (4.4 mm), Kiri (5.0 mm), and Polycam (21.4 mm). Despite these differences, statistical analysis revealed no significant differences in accuracy among the top five apps, indicating that high performance is achievable using either built-in mobile cameras or external sensors—emphasizing the importance of software and reconstruction algorithms over hardware alone. (b) Repeatability was generally high, with low variability across repeated scans and minimal impact of operator differences when standard scanning protocols were followed. (c) Visual and practical differences still matter. Even among statistically similar apps, variations in mesh smoothness and marker/surface clarity were observed, all of which (may) affect clinical usability. (d) Future work should validate these findings in clinical settings, including real-patient testing, volume and overlay accuracy assessments, and the application of AI-based methods to improve reconstruction robustness.

In summary, mobile 3D scanning apps—especially SureScan and Structure Sensor—can already deliver clinically acceptable accuracy. However, variability in reconstruction quality highlights the importance of careful app selection and further clinical validation.

## Data Availability

The data generated and/or analyzed during the current study are not publicly available. However, they can be made available from the corresponding author on reasonable request.

## Appendix A. Application Settings

Table A1 summarizes the settings and configurations used for each scanning application in this study:

**Table A1:** Scanning applications: configurations and export settings.

Application	Camera/Technology	Resolution/Settings	Scan Time	Export Format	Version
SureScan 3D	FaceID camera	3 mm resolution	40 s	.ply	1.1.4
Structure Sensor	Depth sensor	VGA depth, medium range	160 s	.obj	2.17.5
Heges	FaceID camera	Range 5, Precision 0.8 mm, Color 0	91 s	.ply	1.8.1
3D Scanner App	TrueDepth camera	Max depth 1 mm	45 s	.obj	1.1.4
Kiri	Photogrammetry	Auto-capture, 63 images avg.	90 s	.obj	3.14.3
Polycam	Photogrammetry	Auto mode, 37 images avg.	74 s	.obj	5.0.11

## Appendix B. Post-Hoc Statistical Test Results

**Table A2:** Pairwise *p*-values from Dunn’s post-hoc test (Holm-corrected) comparing reconstruction errors between applications. Values in bold indicate statistically significant differences (p<0.05).

	3D Scanner	Heges	Kiri	Polycam	Structure Sensor	SureScan
**3D Scanner**	1.000	0.549	1.000	**0.000**	0.549	0.187
**Heges**		1.000	0.547	**0.000**	1.000	1.000
**Kiri**			1.000	**0.000**	0.547	0.144
**Polycam**				1.000	**0.000**	**0.000**
**Structure Sensor**					1.000	1.000
**SureScan**						1.000

## References

[1] Weissler J.M., Stern C.S., Schreiber J.E., Amirlak B., Tepper O.M. (2017). The evolution of photography and three-dimensional imaging in plastic surgery. Plast. Reconstr. Surg..

[2] Chae M.P., Rozen W.M., Spychal R.T., Hunter-Smith D.J. (2016). Breast volumetric analysis for aesthetic planning in breast reconstruction: A literature review of techniques. Gland. Surg..

[3] Losken A., Seify H., Denson D.D., Paredes A.A., Carlson G.W. (2005). Validating three-dimensional imaging of the breast. Ann. Plast. Surg..

[4] Alshehri S.A., Singh S.K., Mosahebi A., Kalaskar D.M. (2021). The current progress and critical analysis of three-dimensional scanning and three-dimensional printing applications in breast surgery. BJS Open.

[5] Tong O.L.H., Chamson-Reig A., Yip L.C.M., Brackstone M., Diop M., Carson J.J.L. (2020). Structured-light surface scanning system to evaluate breast morphology in standing and supine positions. Sci. Rep..

[6] Akan B., Akan E., Şahan A.O., Kalak M. (2021). Evaluation of 3D Face-Scan images obtained by stereophotogrammetry and smartphone camera. Int. Orthod..

[7] Oranges C.M., Madduri S., Brantner P., Msallem B., Giordano S., Benitez B., Kalbermatten D.F., Schaefer D.J., Thieringer F.M. (2019). Three-dimensional assessment of the breast: Validation of a novel, simple and inexpensive scanning process. In Vivo.

[8] Koban K.C., Härtnagl F., Titze V., Schenck T.L., Giunta R.E. (2018). Chances and limitations of a low-cost mobile 3D scanner for breast imaging in comparison to an established 3D photogrammetric system. J. Plast. Reconstr. Aesthetic Surg..

[9] Behrens A.S., Huebner H., Häberle L., Stamminger M., Zint D., Heindl F., Emons J., Hack C.C., Nabieva N., Uder M. (2025). Comparative assessment of breast volume using a smartphone device versus MRI. Breast Cancer.

[10] Kyriazidis I., Berner J.E., Waked K., Hamdi M. (2025). 3D Breast Scanning in Plastic Surgery Utilizing Free iPhone LiDAR Application: Evaluation, Potential, and Limitations. Aesthetic Surg. J..

[11] Mescheder L., Oechsle M., Niemeyer M., Nowozin S., Geiger A. Occupancy networks: Learning 3D reconstruction in function space. Proceedings of the IEEE/CVF Conference on Computer Vision and Pattern Recognition.

[12] Chang A.X., Funkhouser T., Guibas L., Hanrahan P., Huang Q., Li Z., Savarese S., Savva M., Song S., Su H. (2015). Shapenet: An information-rich 3D model repository. arXiv.

[13] Saito S., Huang Z., Natsume R., Morishima S., Li H., Kanazawa A. Pifu: Pixel-aligned implicit function for high-resolution clothed human digitization. Proceedings of the IEEE/CVF International Conference on Computer Vision.

[14] Kolotouros N., Pavlakos G., Black M., Daniilidis K. Learning to reconstruct 3D human pose and shape via model-fitting in the loop. Proceedings of the IEEE/CVF International Conference on Computer Vision.

[15] Wang N., Zhang Y., Li Z., Fu Y., Liu W., Jiang Y.-G. Pixel2mesh: Generating 3D mesh models from single rgb images. Proceedings of the European Conference on Computer Vision (ECCV).

[16] Weiherer M., von Riedheim A., Brébant V., Egger B., Palm C. (2025). iRBSM: A Deep Implicit 3D Breast Shape Model. Proceedings of the BVM Workshop.

[17] Duarte B., Oliveira B., Torres H.R., Morais P., Fonseca J.C., Vilaça J.L. Robust 3D breast reconstruction based on monocular images and artificial intelligence for robotic guided oncological interventions. Proceedings of the 2023 45th Annual International Conference of the IEEE Engineering in Medicine & Biology Society (EMBC).

[18] Modabber A., Peters F., Brokmeier A., Goloborodko E., Ghassemi A., Lethaus B., Hölzle F., Möhlhenrich S.C. (2016). Influence of connecting two standalone mobile three-dimensional scanners on accuracy comparing with a standard device in facial scanning. J. Oral Maxillofac. Res..

[19] Mikołajczyk M., Kasielska-Trojan A., Antoszewski B. (2019). A new tool for breast anthropometric measurements: Presentation and validation for women and men. Aesthetic Plast. Surg..

[20] Seoni S., Jahmunah V., Salvi M., Barua P.D., Molinari F., Acharya U.R. (2023). Application of uncertainty quantification to artificial intelligence in healthcare: A review of last decade (2013–2023). Comput. Biol. Med..

[21] Lin F., Zakeri A., Xue Y., MacRaild M., Dou H., Zhou Z., Zou Z., Sarrami-Foroushani A., Duan J., Frangi A.F. (2025). From Pixels to Polygons: A Survey of Deep Learning Approaches for Medical Image-to-Mesh Reconstruction. arXiv.

[22] Amiranashvili T., Lüdke D., Li H.B., Zachow S., Menze B.H. (2024). Learning continuous shape priors from sparse data with neural implicit functions. Med. Image Anal..

[23] Shao H.C., Mengke T., Deng J., Zhang Y. (2024). 3D cine-magnetic resonance imaging using spatial and temporal implicit neural representation learning (STINR-MR). Phys. Med. Biol..

[24] Sarasua I., Pölsterl S., Wachinger C. (2021). Transformesh: A transformer network for longitudinal modeling of anatomical meshes. Proceedings of the International Workshop on Machine Learning in Medical Imaging.

[25] Corona-Figueroa A., Frawley J., Taylor S.B., Bethapudi S., Shum H.P.H., Willcocks C.G. Mednerf: Medical neural radiance fields for reconstructing 3D-aware ct-projections from a single x-ray. Proceedings of the 2022 44th Annual International Conference of the IEEE Engineering in Medicine & Biology Society (EMBC).

[26] Vavourakis V., Eiben B., Hipwell J.H., Williams N.R., Keshtgar M., Hawkes D.J. (2016). Multiscale mechano-biological finite element modelling of oncoplastic breast surgery—Numerical study towards surgical planning and cosmetic outcome prediction. PLoS ONE.

[27] Tenajas R., Miraut D., Illana C.I., Alonso-Gonzalez R., Arias-Valcayo F., Herraiz J.L. (2023). Recent advances in artificial intelligence-assisted ultrasound scanning. Appl. Sci..

[28] Murillo-Ortiz B., Hernández-Ramírez A., Rivera-Villanueva T., Suárez-García D., Murguía-Pérez M., Martínez-Garza S., Rodríguez-Penin A., Romero-Coripuna R., López-Partida X.M. (2020). Monofrequency electrical impedance mammography (EIM) diagnostic system in breast cancer screening. BMC Cancer.

[29] Zuluaga-Gomez J., Zerhouni N., Al Masry Z., Devall C., Varnier C. (2019). A survey of breast cancer screening techniques: Thermography and electrical impedance tomography. J. Med. Eng. Technol..

